# Mobile app impulsive buying: A situational factors dataset analysis

**DOI:** 10.1016/j.dib.2023.109559

**Published:** 2023-09-10

**Authors:** Santi Budiman, Tony Wijaya

**Affiliations:** a STIE IEU Yogyakarta, JL. Hayam Wuruk No 20, Yogyakarta 55212, Indonesia; b Universitas Negeri Yogyakarta. Jl. Colombo No 1, Yogyakarta 55281, Indonesia

**Keywords:** Physical environment, Social environment, Time perspective, Hedonic, Utilitarian

## Abstract

This dataset examines the empirical model of situational factors for impulsive buying on mobile apps. The model presents factors driving impulse buying based on situational factors consisting of the physical environment, social environment, time perspective, and hedonic and utilitarian exploration. The data collection technique in this study used a questionnaire distributed online according to predetermined criteria, namely mobile device users who accessed online market board applications and made regular purchases in the last six months. The data were tested statistically using Structural Equation Modeling to ensure the model's fit.

Specifications Table

**Table:** 

Subject	Business, Management, and decision sciences.

Specific subject area	Marketing
Data format	Raw and analyzed.
Type of data	Excel Spreadsheet and Tables.
Data collection	Data were obtained with a survey through a questionnaire regarding the effect of physical environment, social environment, time perspective, and hedonic-utilitarian browsing on mobile apps impulse buying.
Data source location	The data was obtained on the Indonesian online market using an online survey.
Data accessibility	Repository name: Data identification number: DOI: 10.17632/njvcdh6mkz.1 Direct URL to data: https://data.mendeley.com/datasets/njvcdh6mkz/1

## 1 Value of the Data

• These datasets provide valuable insight into the critical factors for predicting the driving factors of impulse buying on mobile apps, which consists of the physical environment, social environment, time perspective, and hedonic and utilitarian browsing. The data meets the model eligibility rules to describe the suitability of empirical conditions and the research model. The data provides an overview of empirical conditions for understanding impulse buying on mobile apps. This data survey can be evaluated by replicating research in other cultures or regions.
• This data also provides essential information on digital marketers considering appropriate online marketing communication programs. Understanding the driving factors of impulse buying on mobile apps can provide relevant information for digital marketing practitioners.
• This data is valuable for researchers interested in digital marketing on mobile apps. Model test data provide evidence of a model that can be developed or replicated for future research focusing on online impulsive buying.

## 2 Data Description

These datasets provide the critical factor for predicting the driving factors of impulse buying on mobile apps, which consists of physical environment, social environment, time perspective, and hedonic and utilitarian browsing. The data were collected using a questionnaire, available as a supplementary file adapted from [1,2,3] and stored in the Mendeley repository. The data contains the results of respondents' statements with a scale of 1-5 from strongly disagree to agree strongly. Each indicator represents each variable with a symbol: PE (physical environment), which consists of 4 items, namely PE1, PE2, PE3, and PE4, SE (social environment) which consists of 3 items namely SE1, SE2, and SE3, TP (time perspective) which consists of 3 items namely TP1, TP2, and TP3, HB (hedonic browsing) which consists of 3 items namely HB1, HB2, and HB3, UB (utilitarian browsing) which consists of 5 items namely UB1, UB2, UB3, UB4 and UB5, and (IB) impulse buying which consists of 3 items namely IB1, IB2, and IB3. A summary of each variable measurement data is presented in Table 1. Structural equation modelling ensures that the data meets the rules of fit or is appropriate based on the goodness of fit indicator. The data regarding the sample demographics are presented in Table 2. The data were analyzed through the data quality test stage. The manifest variable data fulfils the normality test by having a critical ratio below ± 2.58. The data were also tested by using the multivariate outlier data using limit 4 [4]. Based on the multivariate outliers test, it is known that there are no outliers in all variables indicated by the Mahalanobis distance value, which is available as a supplementary file. Table 3 presents the factor load value measured from the latent variable through each observed variable. In making decisions regarding the suitability of latent variables with the observed variables, the minimum factor loading value criteria are set at 0.5 [5]. Overall, the factor loading value of all observed variables from the latent variables is valid and meets the methodological fit measurement model criteria. Reliability is needed to measure the internal consistency of indicators of a construct. Tests of the instrument are carried out using composite reliability and variance extracted. Table 3 shows that the composite reliability coefficient for each variable is above the acceptance value of the reliability limit, namely the minimum value of 0.7 [4]. The value of variant extraction is at the acceptance limit level, a minimum value of 0.5. The test data for the variable measurement model are presented in Table 4. Based on the measurement model testing, the Chi-square, CMIN/df, RMSEA, GFI, TLI, and CFI values meet the required values.

**Table 1:** Construct and measurement.

Construct	Items	Coding
**Physical environment (PE)** PE1 PE2 PE3 PE4	The online marketplace board app that I use is convenient The online marketplace board app that I use features a visually pleasing design The online marketplace board app that I use is visually appealing The features used in the platform are easy to understand	Likert 1-5
**Social environtment (SE)** SE1 SE2 SE3	Most of my friends think using an online marketplace board app is a good idea Almost all of my friends think that we should use an online marketplace application Almost all of my friends recommend me to use an online marketplace application	Likert 1-5
**Time perspective (TP)** TP1 TP2 TP3	I find using the online marketplace board app handy, causing no trouble no matter where I am I feel that using an online marketplace board app does not cause me any problems outside of my home or at work I feel comfortable using the online marketplace board app wherever I am	Likert 1-5
**Hedonic browsing (HB)** HB1 HB2 HB3	When I feel like I can forget about my troubles while browsing online marketplace board apps I feel like I can enjoy my down time while browsing online marketplace board apps I had a lot of fun when I browsed online marketplace board applications	Likert 1-5
**Utilitarian browsing (UB)** UB1 UB2 UB3 UB4 UB5	I browsed online marketplace board apps to buy better priced items I browsed online marketplace board apps to buy better quality items I browsed online marketplace board applications to gather information about products I browsed online marketplace board apps to compare stores I browse online marketplace board apps for more efficient online shopping	Likert 1-5
**Impulse buying (IB)** IB1 IB2 IB3	I have an urge to purchase additional items beyond my shopping purpose when browsing I have an urge to buy something unrelated to my shopping purpose when browsing I have a tendency to buy things outside of my shopping goals when browsing	Likert 1-5

**Table 2:** Sample demographics.

Age (Year)	Women	Men	Total (%)
26-30	93	61	154 (24.5)
31-35	214	172	386 (61.5)
36-40	62	26	88 (14)
Total	369	259	628 (100%)

**Table 3:** Validity and reliability testing.

Variable	λ_i_	ε_i_	Composite reliability
**Physical environment:**			0.74
PE1	0.62	0.38	
PE2	0.80	0.64	
PE3	0.75	0.56	
PE4	0.81	0.65	
**Social environment:**			0.84
SE1	0.78	0.61	
SE2	0.87	0.76	
SE3	0.88	0.77	
**Time perspective:**			0.73
TP1	0.74	0.55	
TP2	0.79	0.62	
TP3	0.80	0.64	
**Utilitarian browsing**:			0.72
UB1	0.77	0.59	
UB2	0.62	0.38	
UB3	0.71	0.50	
UB4	0.64	0.41	
UB5	0.68	0.46	
**Hedonic browsing:**			0.86
HB1	0.86	0.74	
HB2	0.87	0.76	
HB3	0.85	0.72	
**Impulsive buying:**			0.88
IB1	0.87	0.76	
IB2	0.90	0.81	
IB3	0.84	0.71

**Table 4:** Variable's Goodness of fit.

Variable	χ^2^ (∼0)	P (≥0,05)	df	CMIN/Df (≤2)	RMSEA (≤0,08)	GFI (≥0,90)	TLI (≥0,95)	CFI (≥0,95)	Evaluation
Physical environment	1.129	0.971	2	0.003	0.000	1.000	1.000	1.000	Good
Social environtment	2.890	0.343	2	1.945	0.043	0.996	0.997	0.999	Good
Time perspective	3.002	0.335	2	2.001	0.044	0.996	0.997	0.999	Good
Utilitarian browsing	3.285	0.516	2	0.642	0.000	0.999	1.003	1.000	Good
Hedonic browsing	2.517	0.572	2	0.258	0.000	0.999	1.003	1.000	Good
Impulsive buying	Perfect fit								Perfect

## 3 Experimental Design, Materials and Methods

The data were collected using a questionnaire with a Likert scale. The questionnaire was adapted from previous research [1], [2], [3] by conducting a pilot test with as many as 60 respondents or consumers who are used to making online purchases to make the instrument feasible. Structural Equation Modeling is used to measure the acceptance of the empirical model. Structural equation modelling has the advantage of integrating factor analysis with path analysis [4]. The samples collected were 628 respondents from several online platforms in Indonesia. The sampling technique uses non-probability with purposive sampling method, with the criteria of Indonesian consumers aged 18-40 years who were considered to have mobile devices. Data was collected for six months, from April to October 2022, and transformed into an Excel spreadsheet. Data was collected by paying attention to the ethical principles of data collection. Respondents have explained the purpose of data collection. Respondent data is also kept confidential for the privacy of data sources. Data were processed statistically, assisted by the AMOS.

## Limitations

Not applicable.

## Ethics Statement

The authors declare that there are no ethical issues with the data presented. This dataset comprises many online surveys from numerous online consumers, making it unfeasible to obtain individual informed consent. Their participation was voluntary. Therefore, following the ethical guidelines of the Association of Internet Researchers [6], we have anonymized all responses in the dataset. All data have been fully anonymized, and no personal information like email addresses was solicited.

## CRediT authorship contribution statement

**Santi Budiman:** Supervision, Conceptualization, Methodology, Investigation, Resources, Data curation. **Tony Wijaya:** Formal analysis, Data curation, Writing – original draft, Writing – review & editing, Software, Visualization.

## Data Availability

Raw Research data (Original data) (Mendeley Data)

## Appendix Supplementary materials

Image, application 1
