# Supplementary material for: Mobile app impulsive buying: A situational factors dataset analysis
Source: Data Brief. 2023 Sep 10;50:109559. doi: 10.1016/j.dib.2023.109559 (PMC10518667; doi:10.1016/j.dib.2023.109559)
Supplement: Supplementary file 1 [file mmc1.docx]

QUESTIONNAIRE

Description:

*We ask for your willingness to answer the questions below honestly. Each list of questions provided 5 alternative answers. You are asked to choose an alternative answer to the ethical attitude related to the statement below by putting a cross (X) in the box provided.*

Information:

| Strongly Disagree | Disagree | Neutral | Agree | Strongly Agree |
| --- | --- | --- | --- | --- |

Before filling in the list of main questions, you are asked to fill in the respondent's data, any data and answers you provide will be kept confidential.

**Ethical statement**

*Before filling out this statement, I agree with the following ethical matters. This research is solely for the benefit of scientific studies and does not use respondents for detrimental purposes. Collecting this data does not put pressure on me either psychologically, socially or economically. I understand that my participation is voluntary and that I am free to withdraw at any time, without giving a reason and without cost. I consciously and willingly give my opinion without any pressure. I understand that I will be given a copy of this consent form. I voluntarily agree to take part in this study.*

Respondent Data

1. Gender : O Male O Female

2. Age (Year) : O 26-30 O 31-35 O 36-40

|  | **Statement** | **Strongly Disagree** | **Disagree** | **Neutral** | **Agree** | **Strongly Agree** |
| --- | --- | --- | --- | --- | --- | --- |
| PE1 | The online marketplace board app that I use is convenient |  |  |  |  |  |
| PE2 | The online marketplace board app that I use features a visually pleasing design |  |  |  |  |  |
| PE3 | The online marketplace board app that I use is visually appealing |  |  |  |  |  |
| PE4 | The features used in the platform are easy to understand |  |  |  |  |  |
| SE1 | Most of my friends think using an online marketplace board app is a good idea |  |  |  |  |  |
| SE2 | Almost all of my friends think that we should use an online marketplace application |  |  |  |  |  |
| SE3 | Almost all of my friends recommend me to use an online marketplace application |  |  |  |  |  |
| TP1 | I find using the online marketplace board app handy, causing no trouble no matter where I am |  |  |  |  |  |
| TP2 | I feel that using an online marketplace board app does not cause me any problems outside of my home or at work |  |  |  |  |  |
| TP3 | I feel comfortable using the online marketplace board app wherever I am |  |  |  |  |  |
| UB1 | I browsed online marketplace board apps to buy better priced items |  |  |  |  |  |
| UB2 | I browsed online marketplace board apps to buy better quality items |  |  |  |  |  |
| UB3 | I browsed online marketplace board applications to gather information about products |  |  |  |  |  |
| UB4 | I browsed online marketplace board apps to compare stores |  |  |  |  |  |
| UB5 | I browse online marketplace board apps for more efficient online shopping |  |  |  |  |  |
| HB1 | When I feel like I can forget about my troubles while browsing online marketplace board apps |  |  |  |  |  |
| HB2 | I feel like I can enjoy my down time while browsing online marketplace board apps |  |  |  |  |  |
| HB3 | I had a lot of fun when I browsed online marketplace board applications |  |  |  |  |  |
| IB1 | I have an urge to purchase additional items beyond my shopping purpose when browsing |  |  |  |  |  |
| IB2 | I have an urge to buy something unrelated to my shopping purpose when browsing |  |  |  |  |  |
| IB3 | I have a tendency to buy things outside of my shopping goals when browsing |  |  |  |  |  |
